# Practical Resistance of *Ostrinia nubilalis* (Lepidoptera: Crambidae) to Cry1F *Bacillus thuringiensis* maize discovered in Nova Scotia, Canada

**DOI:** 10.1038/s41598-019-54263-2

**Published:** 2019-12-03

**Authors:** Jocelyn L. Smith, Yasmine Farhan, Arthur W. Schaafsma

**Affiliations:** Department of Plant Agriculture, Ridgetown Campus, University of Guelph, 120 Main St. E., Ridgetown, ON N0P 2C0 Canada

**Keywords:** Biotechnology, Plant biotechnology

## Abstract

Transgenic maize, *Zea mays* L., modified to express insecticidal proteins from the bacterium *Bacillus thuringiensis* Berliner, was introduced in 1996 to control *Ostrinia nubilalis* Hübner (Lepidoptera: Crambidae), a key maize pest in North America. The high-dose/refuge concept, developed to delay or prevent resistance evolution to this technology, has been exemplified by *O*. *nubilalis* as no cases of practical resistance were identified in >20 years. This study documents the first case of practical resistance to Cry1F Bt maize by *O*. *nubilalis* in North America. Four collections of *O*. *nubilalis* were made from Cry1F maize in Nova Scotia, Canada with unexpected injury (UXI) ranging from 30–70%. Greater survival of UXI collections was observed when larvae were exposed to the highest concentration of 200 ng Cry1F cm^−2^ in diet-overlay bioassays compared to susceptible laboratory colonies. Larvae also fed and survived on Cry1F leaf tissue in 7 d bioassays. A collection from non-Bt maize, 120 km west of the UXI region, also survived 200 ng Cry1F cm^−2^, but was susceptible to Cry1F leaf tissue. Detection of Cry1F-resistant *O*. *nubilalis* in what might be considered an insignificant maize-growing region indicates that a number of preventable causal factors may have been related to inadequate stewardship of Bt maize technology.

## Introduction

Transgenic maize, *Zea mays* L., modified to express insecticidal proteins from the bacterium *Bacillus thuringiensis* Berliner, commonly referred to as Bt maize, has been widely adopted for control of key maize insect pests in North America, since introduced in 1996. In Canada, approximately 85% of maize acres were planted with Bt maize in 2017^1^. In North America, the primary target pest of Bt maize has historically been the European corn borer *Ostrinia nubilalis* Hübner (Lepidoptera: Crambidae). The distribution of *O*. *nubilalis* overlaps with most of the maize-producing regions in the US and Canada^2^. *Ostrinia nubilalis* is a stalk boring pest whose feeding injury in maize results in yield loss, increased infection of secondary pathogens that cause stalk and ear rots, ear drop, stalk breakage, and lodging, which impedes harvest^2^. The cost of *O*. *nubilalis* management and incurred yield losses were estimated to exceed $1 billion USD before the introduction of Bt maize^2^. Adoption of Bt maize for *O*. *nubilalis* control has resulted in widespread suppression of this pest population in North America, providing economic and environmental benefits to both Bt and non-Bt maize producers^3,4^.

The first commercially available Bt maize events, Bt-11 (Syngenta LLC, Research Triangle Park, NC) and MON810 (Monsanto Company, St. Louis, MO) expressing Cry1Ab protein were approved for use in Canada in 1996 and 1997, respectively^5,6^. In 2002, event TC1507 (co-developed by Dow AgroSciences LLC, Indianapolis, IN and Pioneer Hi-Bred International, Johnston, IA) which expresses Cry1F protein was approved in Canada^7^. To delay or prevent resistance to Bt maize, defined as a heritable decrease in susceptibility by the target pest population, insect resistance management (IRM) strategies were developed for implementation by trait providers and growers. The high-dose/refuge (HDR) strategy, which was developed for *O*. *nubilalis*, has been the foundation for IRM in North America^8^ and has been adapted for other target pests of Bt maize. A highly toxic protein will kill nearly all (>99.9%) homozygous susceptible insects and ~95% of heterozygous susceptible insects^9^. A non-Bt refuge is intended to support an unexposed, susceptible portion of the pest population that will mate with rare, resistant survivors from the Bt crop, thereby maintaining low numbers of individuals carrying resistance alleles within the population. Models for single high-dose Bt toxins predicted 15–30 years of durability with implementation of 5–20% refuge^10,11^. The IRM strategy for Bt maize and *O*. *nubilalis* has been considered exemplary, as practical resistance, defined as field-evolved resistance that reduces the efficacy of the Bt crop and has practical implications for pest control^12^, has not been documented in Canada (J.L.S., unpublished data) or the U.S.^13–16^. Bt-resistant *O*. *nubilalis* have only been reported from field collections in North America in two instances to date. A population collected from Minnesota in 2001 survived a diagnostic concentration of Cry1Ab during routine susceptibility monitoring and survived feeding on Cry1Ab leaf tissue; however, larvae were unable to survive on whole plants expressing Cry1Ab^13^. A collection was identified from Iowa in 2004 that exhibited survival at a diagnostic concentration of Cry1F and survived exposure to Cry1F leaf tissue in assays^14^. Neither case resulted in classification as practical resistance because subsequent monitoring in these regions did not detect reduced susceptibility^14^. Practical resistance to Bt maize has evolved in 15 other lepidopteran pests^16^ in such as *Helicoverpa zea* L.^17^, *Spodoptera frugipera* J.E. Smith^18^, and *Striacosta albicosta* Smith^19,20^, and the coleopteran, *Diabrotica* spp. LeConte^21–23^, presumably because the high-dose requirement was not met between Bt events and their target pest.

**Figure 1:**
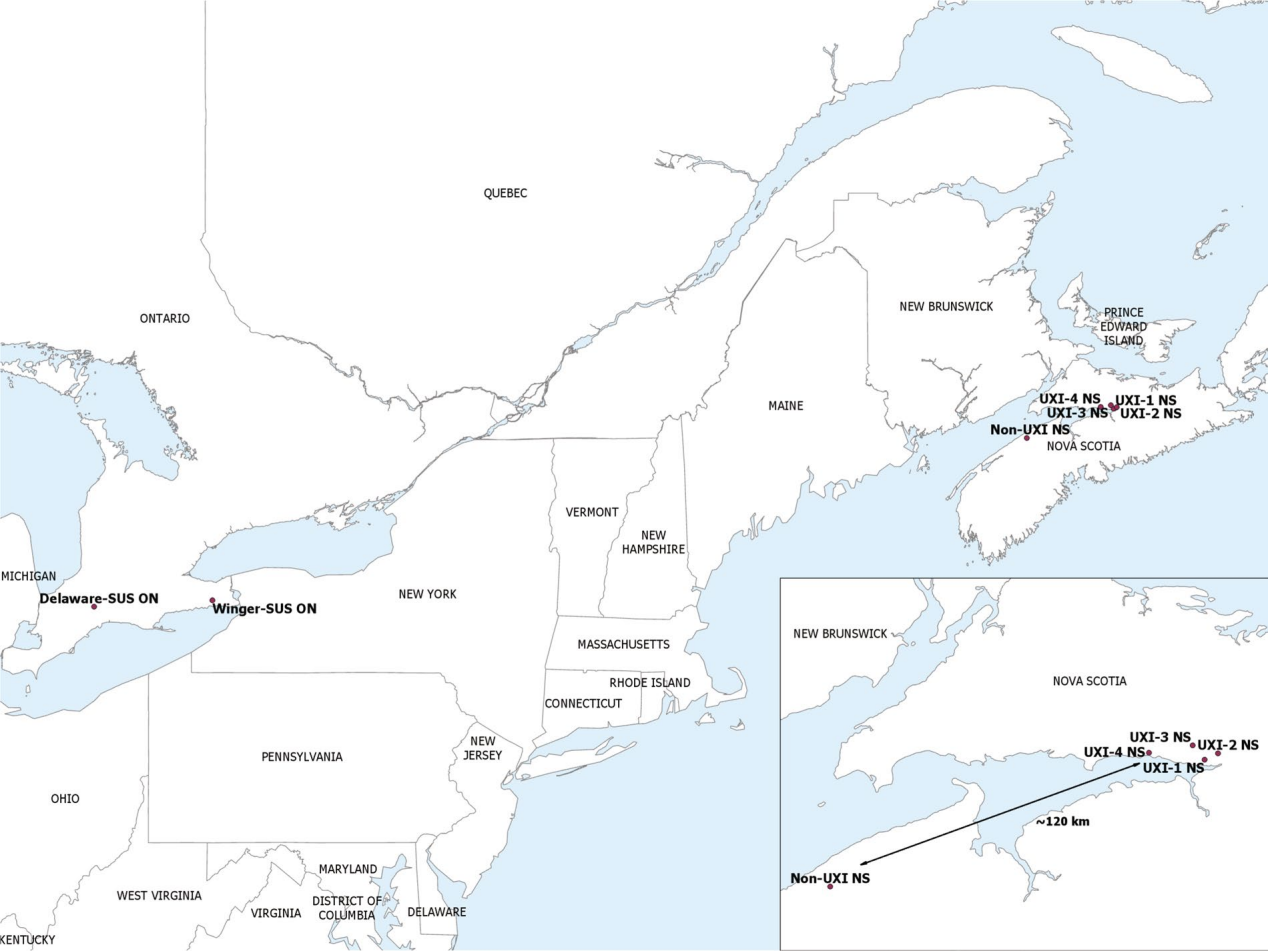
Location of *Ostrinia nubilalis* collections made from four fields with unexpected injury to Cry1F maize hybrids (UXI-1 to UXI-4) and one field of non-Bt maize (Non-UXI) in 2018 in Nova Scotia (NS), Canada. Susceptible laboratory colonies were collected from Winger and Delaware, Ontario (ON) in 2010 and 2016, respectively.

In the fall of 2018, unexpected injury (UXI) by *O*. *nubilalis* to a maize hybrid expressing event TC1507 at a number of fields in Nova Scotia, Canada was reported to the authors, only 12 years after Cry1F hybrids were first marketed in the region. The Maritime Province of Nova Scotia is a predominantly bedrock peninsula surrounded by the Atlantic Ocean (Fig. 1). Nova Scotia is a relatively small maize growing region^24^; approximately 14,000 ha were grown in 2018 (G. Murray, personal communication). Collections of *O*. *nubilalis* were made from four fields with unexpected injury near Truro, NS and one from a non-Bt field approximately 120 km west near Kentville, NS. Second generation offspring of the NS field collections were tested for their susceptibility to Cry1F proteins in diet-overlay concentration response bioassays and to Cry1F and Cry1Ab in leaf tissue bioassays and compared to known susceptible populations of *O*. *nubilalis* collected from the Province of Ontario, Canada. The results of this investigation confirm that *O*. *nubilalis* collected in Nova Scotia are resistant to Cry1F. This is the first case of practical resistance to Cry1F or any Bt protein among *O*. *nubilalis* in North America. Although there are many questions surrounding the development of Cry1F resistance in *O*. *nubilalis* in what might be considered an insignificant maize growing region, a number of causal factors related to inadequate stewardship of Bt maize technology may have been overlooked by stakeholders in that region.

## Results

### Diet bioassays

**Figure 2:**
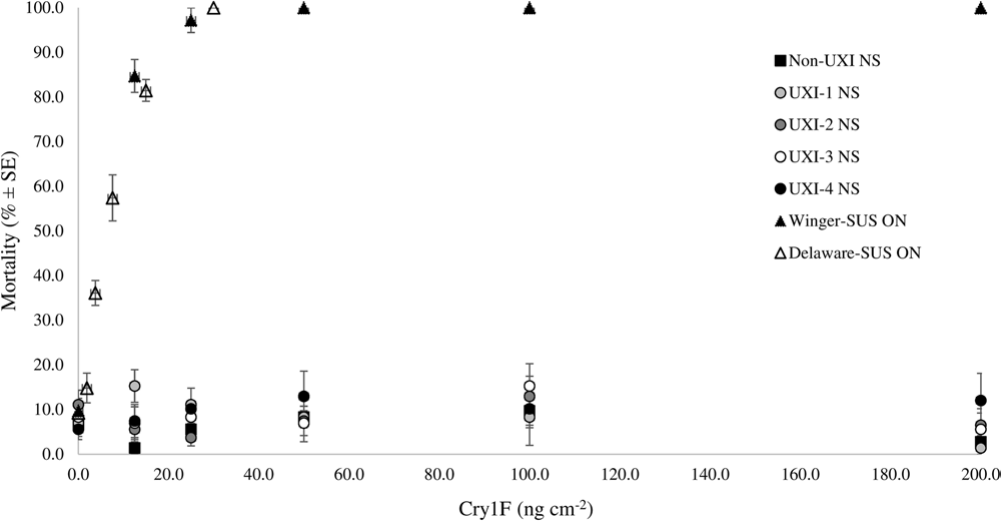
Mortality of neonate (<24 hr old) *Ostrinia nubilalis* exposed to Cry1F protein in diet-overlay bioassays. Larvae were second generation offspring of *O. nubilalis* collected from four fields with unexpected injury to Cry1F maize hybrids (UXI-1 to UXI-4) and one field of non-Bt maize (Non-UXI) in 2018 in Nova Scotia (NS), Canada. Susceptible laboratory colonies were collected from Winger and Delaware, Ontario (ON) in 2010 and 2016, respectively.

Greater than 80% survival was observed among all of the NS UXI collections exposed to the highest concentration of 200 ng Cry1F cm^−2^ 7 days after introduction (DAI) (Fig. 2). Similarly, more than 95% of the larvae from the non-UXI site in NS survived the highest concentration (Fig. 2). Due to the lack of a mortality response within the concentration range tested, lethal concentration values for the UXI collections could not be estimated, but exceeded 200 ng cm^−2^ (Table 1). In contrast, 100% mortality was observed in the Delaware, ON collection at the highest concentration tested (30 ng cm^−2^), and for Winger, ON, 97% mortality was observed at 25 ng cm^−2^ and no survival was observed at any higher concentration (50–200 ng cm^−2^). The mortality responses of the Delaware and Winger, ON collections were alike based on overlapping confidence intervals; LC_50_ values were 6.7 and 6.5 ng cm^−2^, respectively (Table 1).

**Table 1:** Susceptibility and growth inhibition of neonate (<24 hr old) *Ostrinia nubilalis* larvae reared from field collections in Nova Scotia, Canada in 2018 and susceptible laboratory colonies to Cry1F *Bacillus thuringiensis* insecticidal protein in terms of lethal (LC) or effective concentration (EC) with 95% confidence intervals (CI) in brackets.

Collection (Province)	*n*^a^	Slope ± SE	Cry1F (ng/cm^2^)			*Χ*^b^	Cry1F (ng/cm^2^)
			LC_50_ (95% CI)	LC_95_ (95% CI)	LC_99_ (95% CI)		EC_50_ (95% CI)
Winger-Sus (ON)	432	3.31 ± 1.02	6.48 (1.66–9.39)a	20.34 (16.30–35.02)a	32.66 (23.37–108.58)a	6.48	4.43 (2.62–6.01)a
Delaware-Sus (ON)	648	2.84 ± 0.29	6.65 (5.54–7.74)a	25.25 (20.55–33.54)a	43.89 (33.15–65.79)a	12.51	3.78 (2.33–5.93)a
Non-UXI (NS)	432	NE^c^	>200 b	>200 b	>200 b	NE	>200 b
UXI-1 (NS)	432	NE	>200 b	>200 b	>200 b	10.03	>200 b
UXI-2 (NS)	648	NE	>200 b	>200 b	>200 b	24.53*	>200 b
UXI-3 (NS)	432	NE	>200 b	>200 b	>200 b	17.14	>200 b
UXI-4 (NS)	648	NE	>200 b	>200 b	>200 b	23.31*	>200 b

Values followed by the same letter within columns are not significantly different based on overlapping 95% confidence intervals.

^a^Total number of larvae infested in bioassay.

^b^Value of the Chi-square goodness-of-fit test. *p < 0.10.

^c^Not estimable due lack of mortality response within the concentration range tested. Concentrations tested were: 0, 12.5, 25.0, 50.0, 100.0, 200.0 ng Cry1F cm^−2^ for all collections except for Delaware, ON where concentrations tested were: 0, 1.9, 3.8, 7.6, 15.0, 30.0 ng Cry1F cm^−2^.

### Leaf tissue bioassays

**Figure 3:**
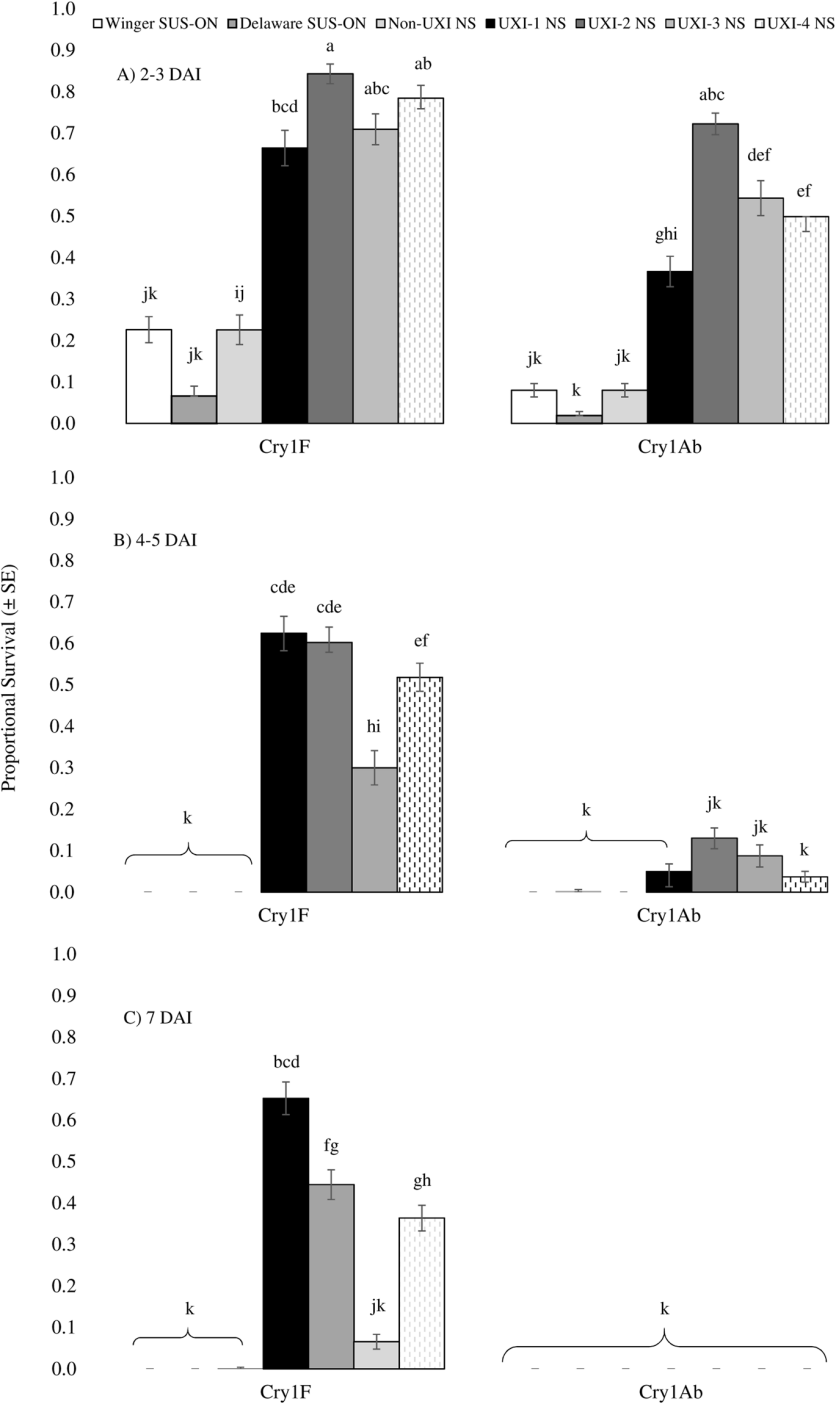
Mean proportional survival ± SE of neonate (<24 hr old) *Ostrinia nubilalis* larvae in Cry1F or Cry1Ab maize leaf tissue bioassays at (**A**) 2–3, (**B**) 4–5, and (**C**) 7 days after introduction (DAI). Larvae were second generation offspring of *O. nubilalis* collected from four fields with unexpected injury to Cry1F maize hybrids (UXI-1 to UXI-4) and one field of non-Bt maize (NON-UXI) in 2018 in Nova Scotia (NS), Canada. Susceptible laboratory colonies were collected from Winger and Delaware, Ontario (ON) in 2010 and 2016, respectively. Means with the same letter are not significantly different (Tukey’s HSD test, *p* > 0.05).

Proportional survival of *O*. *nubilalis* larvae on Cry1F or Cry1Ab leaf tissue depended on the interaction among treatment, collection, and DAI (F_12, 2330_ = 11.31, *p* < 0.0001). At 2–3 DAI, survival of UXI collections on Cry1F tissue ranged from 66–84%; survival of the non-UXI collection, and susceptible Ontario colonies was 23, 7 and 22%, respectively (Fig. 3). Survival on Cry1Ab tissue at 2–3 DAI ranged from 37–72% among UXI collections, 35% for non-UXI, and 2 and 8% of Ontario colonies (Fig. 3). By 4–5 DAI, survival of UXI collections declined to 30–63% on Cry1F tissue (Fig. 2). Neither of the Ontario collections nor the non-UXI collection survived on Cry1F or Cry1Ab tissue beyond 4–5 DAI (Fig. 3). On Cry1Ab tissue, survival among UXI collections ranged from 1–3% at 4–5 DAI. No survival of any collection was observed on Cry1Ab tissue by 7 DAI (Fig. 3). At 7 DAI, 36–66% of larvae from the UXI collections were still alive, except UXD-3 which had <10% survival (Fig. 3).

**Figure 4:**
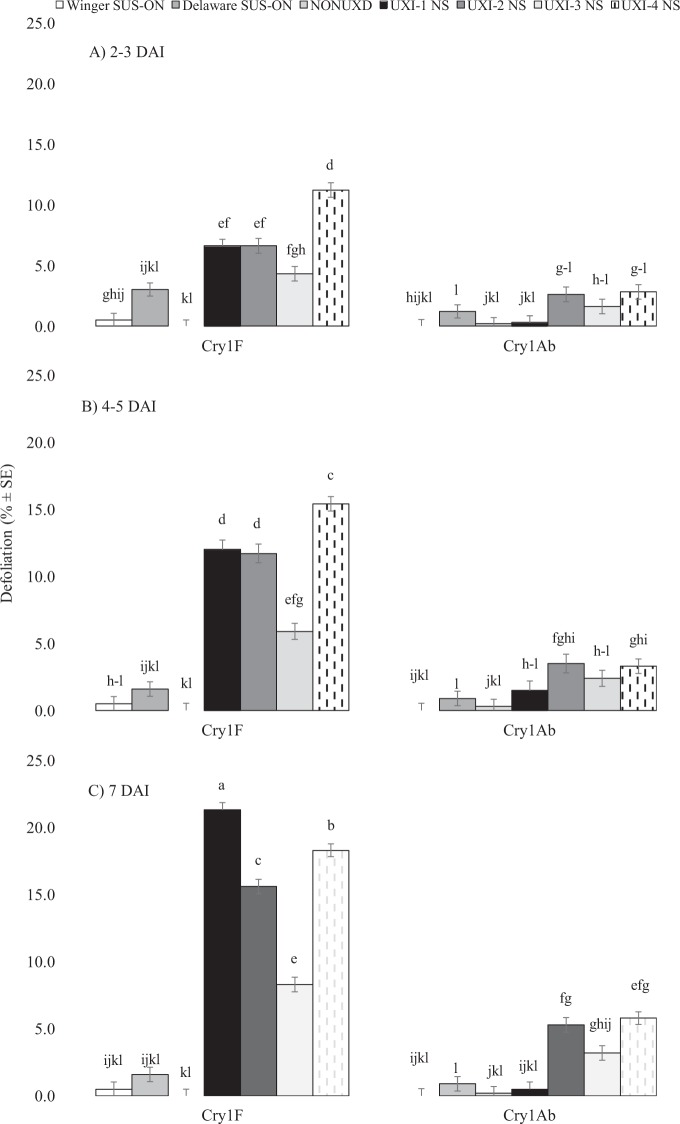
Mean percent defoliation ± SE of Cry1F or Cry1Ab maize leaf tissue by *Ostrinia nubilalis* larvae at (**A**) 2–3, (**B**) 4–5, and (**C**) 7 days after introduction (DAI). Larvae were second generation offspring of *O. nubilalis* collected from four fields with unexpected injury to Cry1F maize hybrids (UXI-1 to UXI-4) and one field of non-Bt maize (NON-UXI) in 2018 in Nova Scotia (NS), Canada. Susceptible laboratory colonies were collected from Winger and Delaware, Ontario (ON) in 2010 and 2016, respectively. Means with the same letter are not significantly different (Tukey’s HSD test, *p* > 0.05).

Percent defoliation of Cry1F and Cry1Ab leaf tissue also depended on the interaction among treatment, collection, and DAI (F_12, 1947_ = 20.44, *p* < 0.0001). On all sampling dates, larvae from UXI collections consumed more leaf tissue than the non-UXI collection and susceptible Ontario colonies, and UXI collections consumed more Cry1F than Cry1Ab leaf tissue (Fig. 4). Larvae from susceptible collections and the non-UXI collection never consumed more than 2% of the Cry1F or Cry1Ab leaf tissue provided. By 7 DAI, larvae from UXI collections had consumed 8–21% of Cry1F leaf tissue (Fig. 4).

## Discussion

This study documents the first case of practical resistance to Bt maize among *O*. *nubilalis* in North America. Observations of Cry1F hybrids with 30–70% injury by *O*. *nubilalis* in Nova Scotia in 2018 and survival of offspring on Cry1F leaf tissue in laboratory bioassays provide evidence that practical resistance to Cry1F has developed among *O*. *nubilalis* in Nova Scotia. Furthermore, offspring of these field collections survived exposure to a concentration of 200 ng Cry1F cm^−2^ in diet-overlay bioassays which is much higher than the diagnostic concentration of 60 ng Cry1F cm^−2^ for Cry1F resistance, derived from extensive testing of U.S. *O*. *nubilalis* populations^25,26^. There are no previously reported data on the susceptibility of *O*. *nubilalis* in Nova Scotia to any Bt proteins. The lack of baseline susceptibility data from this region highlights the limitations of historical susceptibility monitoring efforts and the ability to identify field-evolved resistance; however, unexpected injury to Bt maize has not been previously reported in this region. Nova Scotia populations of *O*. *nubilalis* collected in 2018 were compared with those collected in Ontario in 2010 and 2016 that have been maintained as laboratory colonies shown to be susceptible to Cry1F protein in this study. Since LC_50_ values for Cry1F could not be estimated for Nova Scotia *O*. *nubilalis*, resistance ratios (RR) of LC_50_ values of Nova Scotia and susceptible laboratory colonies could not be calculated. A pest population is generally considered resistant when its LC_50_ value is 10 times greater than the LC_50_ value of a comparable susceptible population^27^. In this study, the LC_50_ values of Nova Scotia populations would have needed to exceed 51–65 ng Cry1F cm^−2^ for a RR >10 which is plausible given the greatest mean mortality observed at 200 ng cm^−2^ was 18.5%.

In addition to requiring a high-dose and non-Bt refuge, the HDR strategy relies on a number of assumptions, including that the frequency of resistance alleles within a population are low (<10^−3^), that resistance is recessive, and that random mating occurs between susceptible and resistant insects^28^. F_1_ and F_2_ screening of *O*. *nubilalis* from the Midwestern U.S. from 2003 to 2009 determined that Cry1F resistance allele frequencies were higher than 10^−3^ ^26^. As allele frequencies did not change significantly during the years of that study, it was suggested that this may have reflected the frequency of alleles within *O*. *nubilalis* populations before the introduction of Cry1F maize^26^. In contrast, allele frequencies for Cry1Ab resistance were found to be very rare among *O*. *nubilalis* collected in France and the U.S.^29^. In laboratory selection experiments, a Cry1F-resistant colony of *O*. *nubilalis* was developed with 3000-fold resistance after 35 generations of selection^25^. Experiments with the laboratory-selected colony and the Iowa field collection showed that a high level of Cry1F resistance was controlled by an autosomal, single locus, recessive factor with weak fitness costs^30,31^.

The results of this study indicate that the four UXI collections from the Truro, NS region have developed greater tolerance to Cry1F than the non-UXI collection taken from non-Bt maize near Kentville, NS. Although all NS collections exhibited tolerance to Cry1F at 200 ng cm^−2^ in diet-overlay bioassays, the UXI collections had greater survival and caused greater defoliation than the non-UXI collection in leaf tissue bioassays. Similarly, the laboratory-selected Cry1F-resistant strain of *O*. *nubilalis* described by Pereira *et al*.^31^ was able to survive concentrations of >12,000 ng Cry1F cm^−2^ in diet-overlay bioassays^25^; however, survival on vegetative Cry1F leaf tissue was approximately 20% lower than on non-Bt isoline tissue. Our leaf tissue bioassay results demonstrated variability in susceptibility among collections from UXI fields which suggests that three of the UXI collections may be homozygous resistant to Cry1F and that the UXI-3 and non-UXI collections may remain heterozygous susceptible, as the dose of Cry1F expressed in leaf tissue has been shown to be high enough to kill or significantly inhibit the development of heterozygotes^31^. Variability of Cry1F expression among maize tissues^32^ may also impact the development and detection of resistance as the dose expressed in vegetative stage plants may be higher than in reproductive stage plants, as Pereira *et al*.^31^ observed greater survival of Cry1F-resistant larvae on R1 stage maize than vegetative stage maize. A short-coming of our study is that a non-Bt near-isoline was unavailable for use as a negative control in the leaf tissue bioassay. Without collaboration with Bt crop developers, public researchers are restricted to using commercially available corn hybrids of which Bt and non-Bt isolines are very rare. Moreover, testing of *O*. *nubilalis* collected in this study against the Bt crop developers’ Cry1F protein was not permitted, however, we were able to obtain Cry1F protein from a public source.

With violation of the assumption that Cry1F resistance alleles in *O*. *nubilalis* populations are rare, the functionally recessive nature of Cry1F resistance indicates that implementation of refuge is critical to maintaining *O*. *nubilalis* susceptibility to Cry1F by production of susceptible insects to mate with potentially resistant ones^14,31^. Since 2005, industry has moved towards Bt maize products that express a pyramid of two or more high-dose toxins targeting *O*. *nubilalis* to prolong Bt event durability with smaller refuge requirements that are more likely to be implemented by growers^10^. The likelihood of resistance development to a pyramid of high-dose toxins is lower than to a single toxin; however the risk increases when single toxin hybrids are not removed from the market and selection pressure is sustained^33,34^. In the Nova Scotia case, information obtained from maize producers and seed providers in the region where resistant populations have been found, confirm that maize hybrids expressing only Cry1F, rather than a pyramid of toxins targeting *O*. *nubilalis*, were used extensively since 2006 up to and including 2018. Although a 20% structured refuge is typically required for single toxin Bt maize products in Canada^35^, data on compliance with this requirement are not available from the region in question. The Canadian Food Inspection Agency, the federal body responsible for regulation of plants with novel traits (PNT) requires that IRM plans are developed for each approved PNT; however, Bt crop developers are responsible for monitoring grower compliance to IRM requirements^36^. Assuming that the frequency of Cry1F resistance alleles within the Nova Scotia *O*. *nubilalis* populations was similar to that found in U.S. populations^25^, a lack of non-Bt refuge may have facilitated the development of this resistant population.

In addition to the selection pressure placed on *O*. *nubilalis* populations in this region due to the use of a single Bt toxin, the geographic characteristics of this region should be investigated for their contribution to resistance development. The phenology of *O*. *nubilalis* in the Maritimes region of Canada is not well understood. Determination of the pheromone races, voltinism, distribution, and host range of *O*. *nubilalis* populations in this region would provide greater understanding of the historical isolation or mixing of *O*. *nubilalis* under Cry1F selection pressure and the probability of successful mitigation of Cry1F resistance in this region. Using a similar recent case for mitigation of a Cry1F-resistant population of *Diatraea grandiosella* Dyar detected in Arizona^37^, a number of strategies should be implemented immediately in the Maritimes region and in neighbouring provinces and states such as New Brunswick, Prince Edward Island, Quebec, Maine, New York, New Hampshire, and Vermont to reduce the size and spread of Cry1F-resistant *O*. *nubilalis* populations.

The sale and planting of Bt maize hybrids expressing only Cry1F should cease immediately to discourage survival of resistant individuals. Maize producers should only plant hybrids expressing a pyramid of at least two Bt toxins that *O*. *nubilalis* remain susceptible to. Although cross-resistance between Cry1F and Cry1Ab has not been documented previously^25^, Bt hybrids expressing only Cry1Ab, pyramids expressing Cry1Ab and Cry1F, or Cry1Ab and Vip3A, should not be used, to decrease selection pressure against Cry1Ab. Elevated survival of NS UXI collections on Cry1Ab leaf tissue compared to susceptible laboratory colonies and the non-UXI collection at 2–3 DAI was observed in this study; therefore further determination of the susceptibility of the Cry1F-resistant collections to Cry1Ab is needed.

In fields where unexpected injury to Cry1F maize has occurred, maize stalks should be chopped and buried using tillage in the fall to reduce the frequency of resistance alleles in *O*. *nubilalis* populations^38^. Cry1F resistance has not been shown to be associated with strong fitness costs in *O*. *nubilali*s, therefore, survival rates of homozygous susceptible, heterozygous resistant, and homozygous resistant *O*. *nubilalis* from non-Bt plants may not differ significantly^30^. As shown by Pereira *et al*., very few heterozygous resistant *O*. *nubilalis* are able to survive on reproductive stage Cry1F plants^31^; therefore, destruction of a significant source of resistant alleles in the overwintering phase will aid in mitigating the resistant population. Lastly, collections of *O*. *nubilalis* from neighbouring provinces and states should be tested to determine the spread of Cry1F-resistant populations. This case highlights the importance of monitoring Bt susceptibility of *O*. *nubilalis* populations in potentially geographically isolated regions where this technology is deployed, especially where single-toxin maize products are grown. We encourage diligence on the part of regulatory and industry stakeholders to increase monitoring efforts for susceptibility of *O*. *nubilalis* populations and unexpected injury to Bt maize for similar regions, and discontinue the use of non-pyramided Bt-maize products.

## Methods

### Insect collections

Four fields with unexpected injury (UXI) by *O*. *nubilalis* to maize hybrids expressing Cry1F protein were visited by the author in early September 2018 after identification by the grower (Fig. 1, Table 2). Seed sales representatives visited the fields in question during the previous week and confirmed Cry1F expression using QuickStix for Cry1F protein (EnviroLogix, Inc., Portland, ME). Additionally, a non-Bt maize field near Kentville, NS, approximately 120 km west and upwind of the UXI fields, infested with *O*. *nubilalis,* was visited within the same week to make a collection of *O*. *nubilalis* from a site lacking Cry1F exposure (Fig. 1, Table 2). The level of infestation was estimated at each site by counting the number of plants exhibiting symptoms of *O*. *nubilalis* injury within 100 examined; across all sites, plants ranged from the R3 (milk) to R5 (dent) stage^39^ (Table 2). Symptomatic plants were cut into sections using pruning shears and placed into mesh bags and shipped to the laboratory at the University of Guelph in Ridgetown, ON, which were received within 4 days of collection. The expression of Cry1F protein was re-confirmed in leaf tissue from a random subsample of five stalks per field using QuickStix for Cry1F protein; all plants from UXI fields tested positive for Cry1F. Stalks were carefully split, larvae were extracted, and placed into tubs containing meridic diet (Table 2)^40^. Rearing conditions were 26:18 °C, 60% RH, and photoperiod of 16:8 (L: D) h for all life stages. Larvae pupated in a corrugated cardboard ring, and the ring was transferred to a cage (30 cm × 30 cm × 60 cm, mesh roof) at the first sign of adult eclosion. Adults were provided with a water source via paper towel wicking *ad libitum*, and the cage sides were misted with water daily. Wax paper sheets were placed over the cage roof as an oviposition substrate, replaced daily, and placed into a plastic sweater box with moistened paper towel. Colonies of *O*. *nubilalis* originally collected from Winger and Delaware, ON in 2010 and 2016, respectively, and maintained for 95 and 17 generations, respectively, were used as susceptible laboratory controls for all experiments (Table 2).

**Table 2:** Location and collection information of *Ostrinia nubilalis* collected in Nova Scotia in 2018 and Ontario in 2010 and 2016 for use in Bt susceptibility bioassays.

Collection	Nearest town	Planting date	Plants with injury by *O*. *nubilalis* (%)	Collection date	No. larvae used to initiate colony	Generation used in bioassays
**Nova Scotia**						
NON-UXI	Kentville	24 May 2018	60	6 Sept 2018	131	F2
UXI-1	Truro	16 May 2018	70	4 Sept 2018	239	F2
UXI-2	Truro	16 May 2018	30	4 Sept 2018	75	F2
UXI-3	Truro	9 May 2018	40	4 Sept 2018	193	F2
UXI-4	Truro	12 May 2018	30	5 Sept 2018	57	F2
**Ontario Susceptible Laboratory Colonies**						
Winger-Sus	Winger	.	.	22 Nov 2010	41	F95
Delaware-Sus	Delaware	.	.	6 Oct 2016	19	F17

### Diet bioassays

Diet used for bioassays was formulated in the same manner as that used for rearing. One mL of diet per well was dispensed into 128-well bioassay trays (Bio-16, CD International, Pitman, NJ) using a repeater pipette. The diet surface area was 2.0 cm^2^. Wells were covered with transparent, adhesive, ventilated covers (Frontier Agricultural Sciences, Newark, DE) after diet had solidified. Trays were stored at 4 °C until required for bioassays. Lyophilized protein standard containing 100% purity truncated Cry1F was obtained from M. Pusztai-Carey (Case-Western University, Cleveland, OH). The protein standard was stored at −80 °C inside a desiccator (Desi-Vac Container™, Thermo Fisher Scientific, Hampton, NH) containing dessicant (Drierite, W.A. Hammond Drietite Co. Ltd., Xenia, OH). Cry1F protein solutions were diluted in series from the stock solution using 10 mM CAPS (3-cyclohexylamino)-1-propane sulfonic acid) buffer solution with pH adjusted to 10.5 using 10 N NaOH. Five concentrations plus a negative control were used for each bioassay. The concentrations tested for all collections included: 0, 12.5, 25, 50, 100, and 200 ng cm^−2^ except for Delaware-Sus ON where the concentrations tested were: 0, 1.9, 3.8, 7.6, 15.0, and 30.0 ng cm^−2^;  the negative control was treated with 10 mM CAPS buffer solution. The appropriate concentration of Bt protein or control solution was applied to the diet surface of each well in 30 µl aliquots using a repeater pipette.

Trays were repeatedly tilted in all directions to cover the entire diet surface with solution. Trays were left in a fume hood until the solvent component of the solution had evaporated. One unfed, neonate (<24 h old) larva was transferred from the plastic sweater box to each well of the bioassay tray using a fine-point paint brush. Tray covers were replaced following introduction, placed into rearing conditions and covered with cardboard to prevent positive phototaxis and condensation build-up. For each bioassay, 24 or 36 larvae were treated per concentration. Larval mortality and individual larval weight were recorded at 7 DAI. Severely stunted larvae that were alive but weighed less than 0.1 mg were considered dead. Each bioassay was repeated 3 times for each collection.

### Leaf tissue bioassays

The maize hybrid P72-11HR which expressed Cry1F was purchased from Pioneer Hi-Bred (Chatham, ON) and the hybrid HZ3011A which expressed Cry1Ab was purchased from Horizon Seeds (Courtland, ON). A non-Bt near-isoline was not available for either hybrid to use as a negative control. Before germination, Bt expression was verified from each seed lot by testing every 16^th^ seed selected for planting using QuickStix for Cry1F protein. Seeds were commercially pre-treated with insecticide and fungicide treatments; therefore, all seeds were washed following the methods of Gassmann *et al*.^21^ before germination. Seeds were germinated on moistened filter paper in a growth room at 26 °C, 60–70% RH, photoperiod of 16:8 (L:D) h. Once germinated, individual seeds were planted 2 cm deep in nursery plug pots (Stuewe and Sons, Inc., Corvallis, OR) with moistened soil for 7–10 d. At the VE stage, plants were transferred to the greenhouse and transplanted into black 8-L plastic pots containing a moistened soil-less mixture prepared from a 1:1 blend of Juniper Pro-Line all-purpose mix (JVK, St. Catherines, ON) and Gro-Coir #3 (Gro-Bark Limited, Waterloo, ON). Each pot was planted with three seedlings of either treatment and pot placement was randomized under the light bank. Plants were grown in a greenhouse (mean daily T ± SD = 25 ± 3 °C; mean daily RH ± SD 52 ± 10%) under natural light supplemented by high-pressure sodium lamps (400 W S51 Lumalux®, Osram Sylvania, Mississauga, Ontario) placed 120 cm above the greenhouse bench, with each lamp covering 2 m^2^, at a photoperiod of 16:8 h L:D. Two weeks after planting, pots were fertilized weekly with 100 mL of Peters Excel 15-5-15 Cal-Mag Special (JVK, St. Catherines, ON) at a concentration of 4 mg mL^−1^. Individual pots were watered as required.

Maize tissues were collected from the greenhouse on the day of bioassay tray preparation. Wells within a 32-well bioassay tray (CD-32, CD International, Pitman, NJ) were filled with 2.5 mL of 2.5% agar. A 9 cm^2^ piece of filter paper (P5 grade, Fisherbrand™) was placed on the agar, and once fully moistened by the agar solution, a 4 cm^2^ piece of V6 stage maize leaf tissue was placed into each well. Expression of Bt events was confirmed in each leaf used in the assay before tissue was placed in the bioassay trays using QuickStix for Cry1F protein. Sixteen wells were treated with Cry1F or Cry1Ab leaves per replicate. Five, unfed, neonate (<24 hr old) larvae were placed onto the leaf tissue in each well, transferred from the plastic sweater box using a fine-point paint brush. Transparent, adhesive, ventilated lids were placed over each well (Frontier Agricultural Sciences, Newark, DE). Infested trays were placed into rearing conditions for the duration of the experiment and covered with cardboard. New tissue was added to each well every 2–3 d to ensure fresh, unlimited food. For each bioassay, 80 larvae were exposed to each treatment and each bioassay was replicated at least 4 times for each collection. At 2–3, 4–5, and 7 DAI, survival and defoliation were assessed. Survival was assessed by gently prodding larvae with a fine-tipped paint brush; larvae that did not respond with movement were considered dead. The number of larvae surviving at each time point was divided by the initial number of larvae infested to calculate proportional survival. Percent defoliation was assessed visually as the area of leaf tissue consumed by the larvae.

### Statistical analysis

#### Diet bioassays

Only diet bioassays with ≥80% survival in the control were used in the analysis. Mortality data were analyzed to determine LC_50_, LC_95_, and LC_99_ values (concentration of Bt protein that resulted in mortality of 50, 95, and 99% of the individuals in a collection, respectively) with 95% confidence intervals and slope of the concentration-response curve using PROC PROBIT in SAS 9.4 (SAS Institute, Cary, NC). The OPTC option was used which estimated the natural response rate of larvae in the control^41^. Pearson’s chi-square test was used to test the fit of observed values compared to the predicted probit model; results were rejected if *p* < 0.10^42^. Weights of surviving larvae from each concentration were pooled to determine the mean weight per larva and transformed to percent growth inhibition relative to the mean weight of larvae in the control. To determine the EC_50_ (concentration of Bt protein that inhibits growth in 50 of the individuals in a collection) for each collection, growth inhibition data were analyzed by nonlinear regression fitted to a probit model using a SAS program written by D. Travnicek (Department of Biometry, University of Nebraska-Lincoln)^43^. Pairwise comparisons among collections were made between LC_50_ values and significant differences were declared for non-overlapping 95% confidence intervals.

#### Leaf tissue bioassays

The effects of treatment, collection, DAI, and their interactions on proportional survival and percent defoliation were analyzed as a generalized linear mixed model using PROC GLIMMIX in SAS 9.4. Replication, replication by treatment, and well within replication by treatment were considered random effects. Dependent variables were treated as repeated measures as observations were taken from wells within replication by treatment at 2–3, 4–5, and 7 DAI. PROC UNIVARIATE and the Shapiro-Wilk statistic were used to test residuals for normal distribution and studentized residuals were calculated to test for outliers using Lund’s test^42^. Proportional survival and percent defoliation followed a normal distribution and no outliers were detected. Least squares means (LSMEANS) were estimated and pairwise comparisons were made using Tukey-Kramer tests (α = 0.05).

## Data Availability

The datasets generated and/or analysed during the current study are available from the corresponding author on reasonable request.
